# A 64-Year-Old Woman With Molluscum Contagiosum Arising in a Melanocytic Nevus: A Case Report of an Unusual Coexistence

**DOI:** 10.7759/cureus.71646

**Published:** 2024-10-16

**Authors:** Sylvia Jimenez, Reuben P Powell, Liliana Rincon, Cecilia Clement

**Affiliations:** 1 John Sealy School of Medicine, University of Texas Medical Branch, Galveston, USA; 2 Pathology, University of Texas Medical Branch, Galveston, USA

**Keywords:** melanocytic lesion, molluscum, nevus, tropism for keratinocytes, tumor regression

## Abstract

Molluscum contagiosum (MC) virus is a poxvirus that infects epidermal keratinocytes producing cutaneous nodules with characteristic intracytoplasmic inclusions. Intradermal nevus is a benign lesion that typically presents as clusters of melanocytes within the dermal layer of skin. Although both MC and melanocytic nevus are common lesions, MC arising within a melanocytic nevus is a rare event with only a few cases published in the literature. Herein, we report the case of a 64-year-old immunocompetent woman with an MC emerging within an intradermal nevus of the left nasolabial fold. In addition, the disappearance of most dermal melanocytes adjacent to the site of the MC infection was noted. This is a rarely reported event, and it has been suggested that it involves a mechanism of viral oncolysis among others, with potential implications for treatment and a need for further investigation.

## Introduction

Molluscum contagiosum (MC) is a viral skin infection caused by a double-stranded DNA poxvirus; it specifically infects keratinocytes, with characteristic eosinophilic intracytoplasmic inclusions, also known as molluscum bodies [1]. Viral inclusion bodies are abnormal structures formed in the cytoplasm (or nucleus) of the host cell that may represent aggregates of mature virus particles; however, most commonly, they are areas where viral synthesis occurs. The MC virus is transmitted by direct contact with infected skin, which can be sexual, non-sexual, or through autoinoculation, or indirectly via fomites. The infection results in lesions characterized by skin-colored, dome-shaped papules. MC infection primarily affects pediatric patients, sexually active young adults, and immunocompromised people of all ages [2]. In immunocompetent individuals, the infection is usually self-limited while in the setting of immunosuppression, the lesions may be widely disseminated, tend to be persistent, and are refractory to treatment. It appears that the immune system plays a role in the containment of the MC virus infection, as evidenced by the presence of spontaneous visible inflammation of individual MC papules that leads to the resolution of these lesions [2]. Although MC is a common skin infection, it is infrequently associated with other skin conditions [3]. Melanocytic nevus, on the other hand, are benign neoplasms composed of melanocytes, which are derived from the neural crest and migrate during embryogenesis to skin and other ectodermal sites (central nervous system, eyes, and ears). The coexistence of MC with melanocytic nevus and its localization within the nevus is an unusual event [3-6]. Herein, we present a case of an immunocompetent, older adult, with an MC emerging within an intradermal melanocytic nevus in the face. In addition, an almost complete disappearance of melanocytes at the site of the viral infection was noted during the histopathologic examination.

## Case presentation

**Figure 1:**
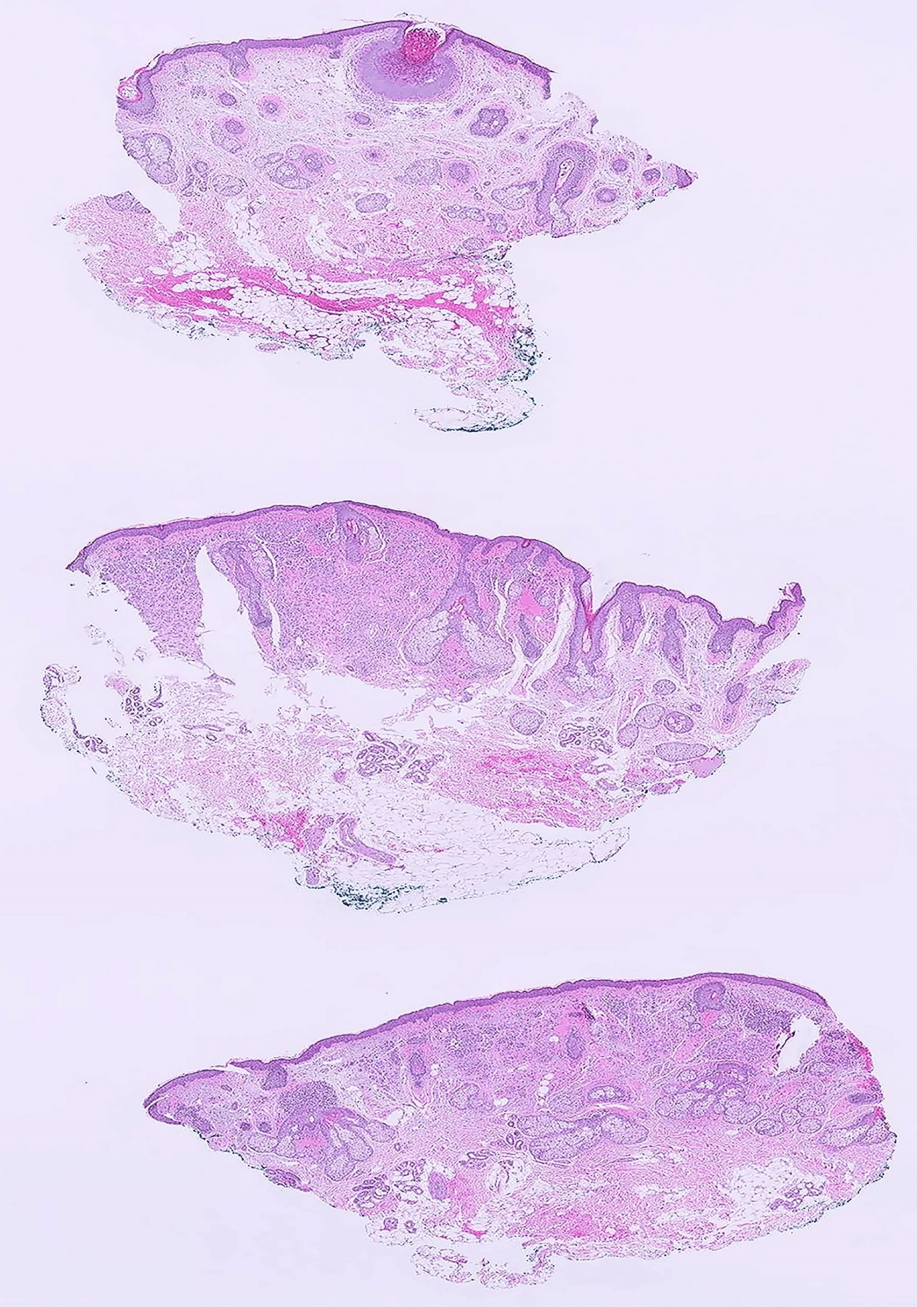
Microscopic examination of the trisected skin ellipse The upper section shows well-circumscribed, cup-shaped, endophytic growth of squamous epithelium. The middle and lower sections show small nests of melanocytes limited to the upper dermis and surrounding adnexa, with an intact epidermal and dermal-epidermal junction (H&E, x10).

A 64-year-old female with a past medical history of anxiety, arthritis, hypertension, and mild hyperlipidemia, presented to the Dermatology clinic for evaluation of a raised lesion of the left nasolabial fold. The lesion had been present for several years and had recently grown. The patient was immunocompetent, with no history of skin cancer, never smoked, and had no history of alcohol use. She was retired and living with her husband. Physical examination revealed a well-circumscribed papule, with mild dark pigmentation, in the left nasolabial fold, which was clinically diagnosed as an intradermal nevus. The patient did not show any other melanocytic lesions, but actinic keratoses in the face, which were treated with cryotherapy during the visit, and a few seborrheic keratoses on her back. The decision to proceed with surgical excision of the nevus was made, due to cosmetic reasons and per the request of the patient, and she was referred to the Plastic Surgery clinic. The excised skin ellipse (1.2 x 0.5 cm) received in pathology showed a 0.5 x 0.5 cm slightly pigmented, centrally located nodule. The specimen was trisected, and histologic sections at low magnification are shown in Figure 1. There was a well-circumscribed, cup-shaped lesion with endophytic growth of the squamous epithelium, limited to the upper section, suggestive of MC. The middle and lower sections revealed small nests of melanocytes limited to the upper dermis and surrounding adnexa, with an intact epidermal and dermal-epidermal junction.

**Figure 2:**
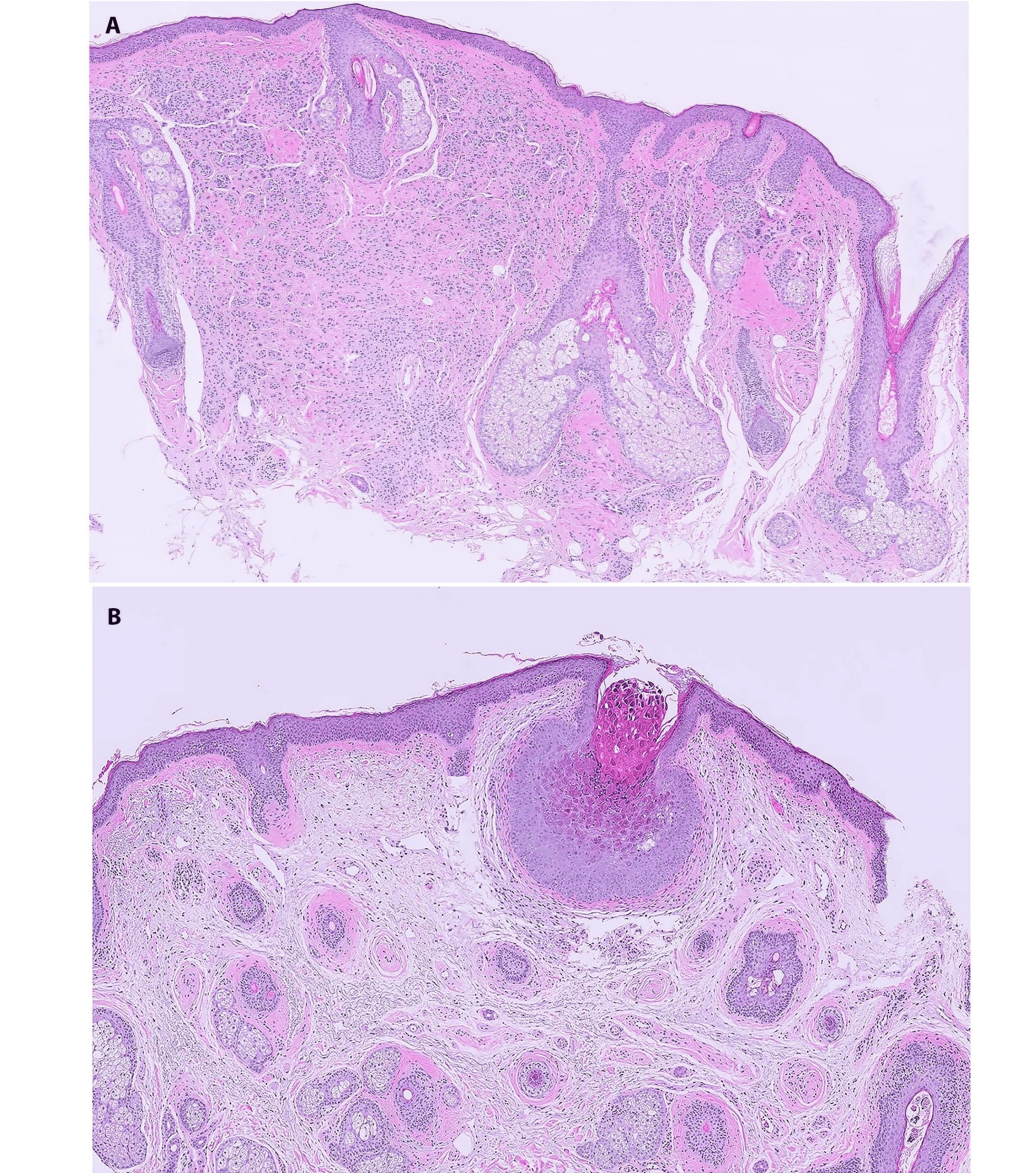
Molluscum contagiosum in an intradermal nevus (A) Intradermal nevus, high power view, showing the characteristic dermal melanocytic proliferation. (B) Normal squamous epithelium flanking a cup-shaped lesion with an inverted lobule of hyperplastic squamous epithelium expanding into the superficial dermis. The epithelial cells show nuclei compressed by eosinophilic intra-cytoplasmic viral inclusions, molluscum bodies, seen at higher magnification. Note the disappearance of most dermal nevus cells in the areas adjacent to the molluscum (H&E, x50).

Examination at high magnification confirmed an intact epidermal and dermal-epidermal junction, with an intradermal proliferation of bland, epithelioid nevus cells, displaying the characteristic histologic maturation of an intradermal type of melanocytic nevus in which the nevus cells become smaller with less cytoplasm as they progressively descend into the dermis (Figure 2A). Figure 2B highlights the cup-shaped proliferation with inverted lobules of hyperplastic squamous epithelium expanded into the underlying dermis and flanked by normal squamous epithelium. The epithelial cells within this endophytic growth contained numerous large eosinophilic-to-basophilic intracytoplasmic viral inclusions and molluscum bodies, consistent with MC. Interestingly, the melanocytes disappeared within the immediate vicinity of the MC, leaving a few residual nevus cells in the dermis below the molluscum.

Mild fibrosis surrounded the MC, but there was no lymphocytic infiltration or proliferation of melanophages, both common features of regression in melanocytic lesions.

The patient tolerated the surgical procedure well and was released home. The lesion was completely excised and no additional treatment was provided.

## Discussion

The association of an MC infection with conditions compromising the epidermal barrier has been described [5-7]. A large case series showed that almost 4% of cases of MC submitted for histopathologic analysis occur in association with another lesion [3]. The most common lesions found in association with MC in this series and other reports were epidermal inclusion cysts (EIC) [3,7]. Phelps et al. described two cases showing different histopathologic features. One case demonstrated MC viral changes in addition to multilocularity throughout the entire EIC lesion, whereas the second case showed a small unilocular cyst with only focal microscopic evidence of MC virus infection. The author suggested two hypotheses to explain the pathogenesis of these lesions. One proposal was the downward extension and cystic change in a preexisting superficial MC virus infection, indicating the virus played a role in the development of EIC and a possible mechanism in Case 1. The second proposed mechanism, explaining Case 2, referred to the invasion of the MC virus into a preexisting EIC [7]. Certainly, the latter is in concordance with the association of MC with conditions where the epidermal barrier is compromised.

Melanocytic lesions, benign or malignant, coexisting with MC is a much less common occurrence [3-6,8,9]. There are few reports of melanocytic nevi with intranuclear inclusion bodies associated with previous MC viral infection, indicating that, despite the specific tropism for keratinocytes, the MC virus may be able to infect melanocytes [10,11]. One possible explanation for the MC arising within a nevus would be the proliferation of melanocytes within the dermis, leading to surface fragility and predisposing to the development of MC [5,6], similar to the previously mentioned association of MC with compromised epidermal barrier conditions. In our patient, the melanocytic lesion being present for several years, and reportedly growing more recently, could agree with this explanation.

Marks et al. described a case of MC occurring in a halo nevus [8]. In that case, the halo nevus had been present for years before MC occurred, demonstrating that MC can colonize preexisting lesions independently of the immune status of the patient. Interestingly, Cribier et al. reported two cases of MC associated with epidermal nevus present since birth, where the diagnosis of MC was not suspected, as in our case. However, the occurrence of rapid growth of the tumor led to the excision of the lesion, which proved to be MC colonizing the hyperkeratotic epidermis [3]. Similarly, Dobrosavljevic et al. described a patient with a nevus on the back of the trunk, present since childhood, which became depigmented and slightly red, and was excised. On histological examination, a dermal-type melanocytic nevus with MC findings on one side was found [4]. Our case is one of few reports raising awareness of this unusual MC-melanocytic lesion coexistence, potentially causing changes in the appearance of the nevus that could raise concern for malignant transformation.

A more intriguing and rarely reported occurrence is the disappearance of melanocytes at the periphery of the coexisting MC [4,9]. Spontaneous regression could be an explanation, as it is a phenomenon commonly seen in melanoma and other melanocytic lesions. Many mechanisms have been described to explain melanocytic tumor regression, but the most consistently associated is an immune response via cytotoxic tumor infiltrate lymphocytes attacking the melanocytic tumor cells and leaving behind a fibrotic scar [12,13]. Histopathologic features that are characteristic of melanocytic nevus regression include the presence of a notable lymphocytic inflammatory infiltrate, melanophages, vascular proliferation, and dermal fibrosis [12]. Given the lack of lymphocytes and macrophages, a strong immune response and spontaneous regression seem less likely explanations for the disappearance of melanocytes in our case. Another proposed mechanism to explain the disappearance of melanocytes adjacent to the molluscum site is the selective destruction of melanocytes by the MC viral infection [4,9]. Dobrosavljevic et al. reported a case of complete destruction of melanocytes and melanoma cells occurring on the site of an MC infection, suggesting the MC virus as a potential candidate for viral oncolysis [4]. The idea of using viruses to induce cancer regression is not new; it has been around since the early 1950s [4]. Oncolytic virotherapy is an emerging novel tumor therapeutic approach that selectively replicates in and destroys tumor cells while leaving normal cells undamaged. Clinical trials of both naturally occurring oncolytic viruses (e.g., reovirus and vesicular stomatitis virus) and genetically engineered oncolytic viruses (e.g., adenovirus, vaccinia virus, and herpesvirus) have been taking place with some encouraging data [14]. Oncolytic viral therapies have been tested for melanoma. One oncolytic virus called T-VEC (modified oncolytic herpes simplex virus 1) has been approved by the Food and Drug Administration (FDA) for the treatment of melanoma [14]. Oncolytic viruses have recently emerged as a standard treatment option for patients with advanced melanoma, with several oncolytic viruses in development. Immuno-oncolytic virotherapy combined with immune checkpoint inhibitors is promising for the treatment of advanced melanoma [15]. In this context, the coexistence of MC and nevi in addition to the described associated melanocyte disappearance is intriguing and reopens the discussion of MC virus utility for oncolytic therapies of melanocytic neoplasms. 

## Conclusions

The clinical diagnosis of MC can be difficult, especially in immunocompetent, older adults, and with localization of the lesion in the head. The diagnosis could be more complicated when MC is colonizing pre-existing lesions, as reported in this case, coexisting with melanocytic nevus, in which the diagnosis of MC was only done by histopathologic examination. This report brings awareness of the potential preferential localization of MC over melanocytic lesions and other cutaneous conditions and considers MC in the differential diagnosis over potential malignant transformation when encountering changes in long-standing nevi. In addition, there are very few reports on the disappearance of dermal melanocytes in melanocytic lesions associated with MC in the literature, and hypotheses about a viral oncolytic effect of MC virus infection have been raised. However, more studies on its potential implications for the treatment of melanocytic neoplasms and their reproducibility remain to be further investigated.
